# Climate Change Adaptation Strategies by Indonesian Vegetable Farmers: Comparative Study of Organic and Conventional Farmers

**DOI:** 10.1155/2022/3590769

**Published:** 2022-04-11

**Authors:** Irham Irham, Irma Audiah Fachrista, Masyhuri Masyhuri, Any Suryantini

**Affiliations:** ^1^Department of Agricultural Socioeconomics, Faculty of Agriculture, Universitas Gadjah Mada, Yogyakarta, Indonesia; ^2^Assessment Institute for Agricultural Technology of Bangka Belitung, Indonesian Agency for Agricultural Research and Development Ministry of Agriculture, Jakarta, Indonesia

## Abstract

Some experts believe that organic agriculture is more adaptable compared to conventional agriculture. Accordingly, the purpose of this study is to assess organic and conventional farmers' perception and adaptation to climate change and analyse the factors that influence such decisions. The survey was conducted in Java, involving 112 organic farmers and 112 conventional farmers. The chi-square test was used to differentiate climate change perceptions and adaptation strategies applied by farmers. The factors that influenced the selection of the adaptation strategies were analysed using logistic regression. The results of analysis found that organic farmers have more precise perceptions of climate change than that of conventional farmers. Organic farmers more commonly implement mixed cropping, crop rotation, increasing organic manure, using shade, and changing irrigation techniques as their adaptation strategies, while conventional farmers more commonly prefer to adjust planting and harvesting dates and use traditional climate prediction called Pranata Mangsa. The selection of farmers' adaptation strategies is influenced by age, education, experience, distance to extension services, access to credit, information about climate and farmer groups, as well as farmers' perceptions of climate change. The results of the study recommend that policy makers increase farmers' adaptive capacity through investment in education and institutions to support climate change adaptation.

## 1. Introduction

Climate change has become a serious threat to agricultural sectors in Indonesia, including horticulture, because it can disrupt sustainability and production systems [1]. Climate change may decrease the quantity and quality of yields, increase pests and diseases outbreak, and result in horticultural crop failure, especially vegetables [2, 3]. Vegetables often require water supplies to increase and improve the quality of yields. For example, soil water shortage in the beginning of shallot cultivation may decrease the production by 26% [4]. Appropriate selection of adaptation strategies is necessary for farmers to reduce the negative impacts of climate change [5–9]. One of the adaptation strategy alternatives is organic farming. Organic farming has higher endurance and adaptation during extreme conditions because organic agriculture uses a higher level of organic manure than conventional agriculture that can improve the water holding capacity of soil, thus decreasing the risk of yield loss; organic agriculture applies various traditional skills and knowledge to manipulate agroecosystems, thus decreasing dependence on external inputs; and organic farming involves plant diversification, rotation, landscapes, and agricultural activities that may increase farmers' resilience in facing climate change impacts [10, 11].

In recent years, research on farmers' perceptions and adaptation to climate change has become a concern for farmers, researchers, and policy makers [12, 13]. Farmers' perception and knowledge in facing climate change will determine the actions, measures, adjustments, and adaptation strategies to climate change in the long term [14]. Previous research on perceptions revealed that awareness and perceptions of climate change vary within and across regions [15–21]. Farmers implement various adaptation strategies to reduce climate change impacts [22–29]. There are several factors which influence farmers' selection of adaptation strategies such as demographic characteristics [29, 30], farming characteristics [31, 32], cognitive and psychological factors [33], accessibility of resources [22], and sociocultural factors [34].

Comparative studies of organic and conventional vegetable farmers on climate change and its impacts, the adaptation measures they take to overcome the situation, and the main factors that affect the selection of adaptation strategies are still limited in Indonesia. The purpose of this research is to assess organic and conventional farmers' perception and adaptation to climate change and analyse the factors that influence such decisions. This study is important because understanding the perceptions of vegetable farmers and the way they think and act in response to climate change is very important in dealing with the negative impacts of climate change and in maintaining the productivity of vegetable farming. In particular, this study seeks to examine the following research questions: what are the perceptions of organic and conventional vegetable farmers about climate change and the negative impact of climate change on vegetable farming? what are the adaptation strategies selected by organic and conventional vegetable farmers to overcome the negative impacts of climate change on vegetable farming? and what are the factors influencing the adaptation strategies selected by organic and conventional vegetable farmers in dealing with climate change?

This article proceeds as follows. We first present in Section 2 the materials and methods. We interviewed 224 farmers and analysed the data using chi-square and logistic regression. Our results are presented in Section 3, in which we provide farmers' socioeconomic characteristics, vegetable farmers' perceptions of climate change and its impacts on vegetable farming, farmers' adaptation strategies in dealing with climate change, and factors influencing vegetable farmers' selection of adaptation strategies in dealing with climate change. A discussion is presented in Section 4.  Section 5 concludes this work.

## 2. Materials and Methods

### 2.1. Study Location

**Figure 1:**
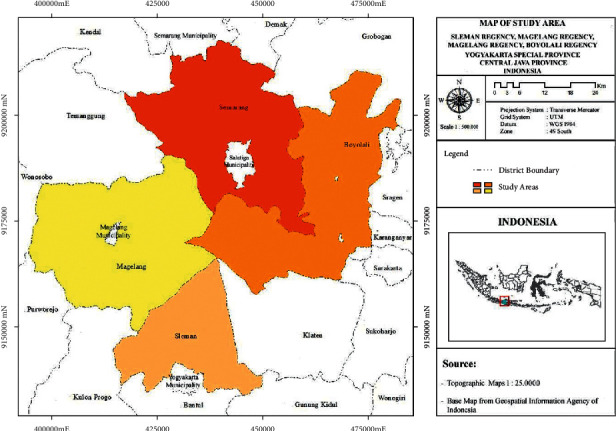
Research sites map.

The study was conducted in Central Java Province and the Special Region of Yogyakarta Province, Indonesia. These two provinces are the locations for the development of smallholder organic farming. Then, four districts where organic vegetables were cultivated were selected deliberately as the research locations, i.e., Sleman (special region of Yogyakarta) and Magelang, Boyolali, and Semarang regencies (Central Java) (Figure 1).

### 2.2. Survey

The survey was conducted in February–August 2018 by interviewing 112 respondents of organic vegetable farmers who were randomly selected, and as a comparison, 112 conventional farmers around the organic farmers were also interviewed. The survey was carried out using a structured questionnaire. The first section of the questionnaire collected information about socioeconomic characteristics including age, education, experience, access to various institutions, and background of vegetable farming activities. The next part investigated farmers' perceptions of climate change and its impacts on vegetable farming. Farmers' perceptions of climate change were assessed based on the indicators of rainfall and temperature over the last 30 years. The last section contained various alternatives of adaptation strategies or changes in vegetable farming practices carried out by farmers in response to perceived climate change.

### 2.3. Analysis

The data were analysed using chi-square and logistic regression. Chi-square was used to determine whether there was a significant difference between organic and conventional farmers' perceptions of climate change and its impacts. Logistic regression was used to find out about the factors influencing the organic and conventional vegetable farmers' selection of adaptation strategies in dealing with climate change. The logistic regression model was considered the right model to use because the dependent variable was binary (whether or not adaptation strategies were used to minimize the negative impacts of climate change; yes = 1, 0 = otherwise). The coefficient of logistic regression analysis could not explain the effect of variable change *X*_*n*_, the probability of farmers to adopt certain adaptation strategies (Pr *Y*_ij_ = 1). Interpretation and measurement of the results may use the marginal effect or partial elasticity. The marginal effect describes the effect of changes in the explanatory variable *X*_*n*_ on the probability of farmers' adaptation strategies. Partial elasticity measures the effect of increasing explanatory variable *X*_*n*_ by 1% on the change in the probability of farmers' adaptation strategies. A study conducted by [5] used the marginal effect when explanatory variable *X*_*n*_ was a binary variable or used partial elasticity when explanatory variable *X*_*n*_ was continuous variable.

The explanatory variables which affected the selection of adaptation strategies (Table 1) were selected based on the findings of previous research; such as age [29, 35, 36], education [25, 29, 35–39], farming experience [36], distance to extension services [31], distance to input market [28, 40], access to credit [41], access to climate information [42], farmer group membership [41, 43], access to climate training [23, 32], and perception of climate change [44].

## 3. Results

### 3.1. Farmers' Socioeconomic Characteristics

In general, the conditions of organic vegetable farmers are relatively better than those of conventional farmers in terms of socioeconomic conditions (Table 2). Organic vegetable farmers have better accessibility to credit institutions and farmer groups membership as well as closer distance to input market than conventional vegetable farmers. In addition, there is also a significant difference in the perceptions of decreased rainfall between organic and conventional vegetable farmers, where more organic farmers believe that rainfall tends to decline over the past 30 years compared to conventional farmers. Nonetheless, there are a few exceptions regarding the socioeconomic conditions, i.e., organic farmers have farther distance to extension services compared to conventional farmers.

### 3.2. Vegetable Farmers' Perceptions of Climate Change and Its Impacts on Vegetable Farming

**Figure 2:**
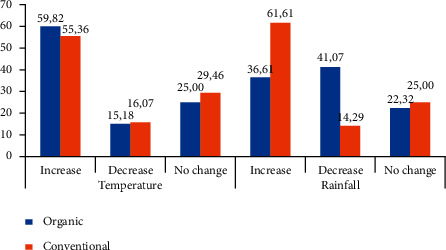
Organic and conventional vegetable farmers' perceptions of changes in rainfall and temperature over the past 30 years in Java, Indonesia, 2018 (% of respondents).

The results showed that most organic and conventional farmers have the same perceptions in terms of temperature, i.e., the temperature increases over the past 30 years. On the other hand, rainfall is perceived differently between organic and conventional farmers, i.e., most conventional farmers perceive that rainfall increases, while organic farmers perceive that rainfall in the research locations decrease over the past 30 years (Figure 2).

Both organic and conventional farmers perceive the negative impacts of climate change on vegetable farming (Table 3). In general, more conventional farmers perceive the negative impacts of climate change compared to organic farmers. Three negative impacts of climate change perceived by most organic and conventional vegetable farmers are an increase in pests and diseases, decrease in the quality of yields, and decrease in production. In addition, an increase in pests and diseases is the most significant impact experienced by conventional farmers compared to organic farmers.

### 3.3. Vegetable Farmers' Adaptation Strategies in Dealing with Climate Change

Both organic and conventional vegetable farmers make efforts to reduce the impacts of climate change by implementing various adaptation strategies in vegetable farming. However, the adaptation strategies implemented by organic vegetable farmers are more varied than those by conventional farmers (Table 4). Organic vegetable farmers have more adaptation strategies compared to conventional vegetable farmers, including mixed cropping, crop rotation, increasing the use of organic manure, using shade, and changing irrigation techniques. On the other hand, compared to organic farmers, there are more conventional vegetable farmers who implement other adaptation strategies such as growing nonwater intensive crops, adjusting planting, and harvesting dates, and using Pranata Mangsa (traditional planting season calendar) to minimize the negative impacts of climate change.

### 3.4. Factors Influencing Vegetable Farmers' Selection of Adaptation Strategies in Dealing with Climate Change

The values of marginal and partial elasticity coefficient resulted from the analysis of the factors that influence the strategies selected by organic vegetable farmers are given in Table 5, while the values of marginal and partial elasticity coefficient resulted from the analysis of the factors that influence the strategies selected by conventional vegetable farmers are given in Table 6. The results showed that age, education, experience, distance to extension services, access to credit, information of climate, and farmer groups as well as farmers' perceptions of temperature and rainfall changes over the past 30 years influence the climate change adaptation strategies selected by both organic and conventional farmers. The effects of each of these variables on both organic and conventional farmers' selection of adaptation strategies are as follows.

#### 3.4.1. Age

The coefficient of farmers' age resulted in a negative sign on the significant selection of adaptation strategies, indicating that age negatively influences the probability of adaptation strategy selected by farmers. For example, the fact that age has a negative effect on Pranata Mangsa (traditional planting season calendar) strategy implemented by organic farmers indicates that an increase in age significantly decreases the probability of organic farmers to use Pranata Mangsa adaptation strategy. The value of partial elasticity shows that an increase in farmer's age by 1% will decrease the probability of farmers to select Pranata Mangsa adaptation strategy by 0.65%. In addition, among conventional farmers, an increase in age by 1% will lower the probability of farmers to implement mixed cropping, grow nonwater intensive crops, conduct crop rotations, and adjust planting and harvesting dates by 0.68%, 0.21%, 0.39%, and 0.49%, respectively.

#### 3.4.2. Education

Farmers' education resulted in either positive or negative signs on various adaptation strategies that farmers select, meaning that an increase in the length of education undergone by farmers may increase or decrease the probability of farmers to select a particular adaptation strategy. A positive effect of education on the probability of farmers in selecting an adaptation strategy can be seen in a strategy to increase the use of organic manure and the use of shade, indicating that an increase in the length of education by 1% among organic farmers will increase the probability of organic farmers to increase the use of organic manure and shade by 0.55% and 0.68%, respectively, while such increase among conventional farmers will increase the probability of using superior varieties by 0.18%. On the other hand, a negative effect of education on some adaptation strategies selected by organic farmers can be seen in the strategy to grow nonwater intensive crops (0.03%), adjust planting and harvesting dates (0.69%), and use Pranata Mangsa (0.87%), while in conventional farmers, it can be seen in the adaptation strategies to adjust planting and harvesting dates (0.46%), increase the dose of organic manure (0.31%), and use of Pranata Mangsa (0.87%).

#### 3.4.3. Farming Experience

Farmers' experience in vegetable farming is positively related to several adaptation strategies implemented by organic and conventional farmers. For example, there is a positive relationship between farming experience and the implementation of Pranata Mangsa adaptation strategy, meaning that an increase in organic farmers' experience by 1% increases the probability of farmers to select Pranata Mangsa adaptation strategy by 0.03%. Among conventional farmers, an increase in farming experience by 1% increases the probability of farmers to implement mixed cropping adaptation strategy (0.28%), use superior varieties (0.32%), conduct crop rotation (0.23%), and use Pranata Mangsa (0.52%).

#### 3.4.4. Distance to Extension Services

Distance to extension services has a negative effect on several adaptation strategies implemented by organic and conventional farmers. Among organic farmers, closer distance to extension services by 1% increases the probability of farmers to use superior varieties (0.08%) and adjust planting and harvesting dates (0.22%). Among conventional farmers, a closer distance to extension services by 1% increases the probability of conventional farmers to implement mixed cropping (0.29%), conduct crop rotation (0.19%), and use shade (0.62%).

#### 3.4.5. Distance to Input Markets

Distance to input market is negatively related to several adaptation strategies selected by organic and conventional farmers. Farther distance to input markets increases the probability of farmers to implement mixed cropping (0.22%), grow nonwater intensive crops (0.48%), conduct crop rotation (0.22%), adjust planting and harvesting dates (0.31%), use shade (0.56%), and use Pranata Mangsa (0.39%). Among conventional farmers, closer distance to input market by 1% increases the probability to implement several adaptation strategies such as mixed cropping (0.23%), crop rotation (0.14%), adjust planting and harvesting dates (0.42%), and use mulch (0.1%).

#### 3.4.6. Access to Credit

Access to agricultural credit has a positive effect on the strategy to increase the use of organic manure by organic farmers and the use of mulch by conventional farmers. Farmers who have access to credit have a higher probability to implement the adaptation strategies of using organic manure and mulch by 25.3% and 11%, respectively, compared to conventional farmers.

#### 3.4.7. Access to Climate Information

Climate information has a positive effect on all the significant adaptation strategies selected. Climate information increases the probability of organic farmers to implement several adaptation strategies including mixed cropping (10.6%), crop rotation (10.6%), adjust planting and harvesting dates (26.6%), and use Pranata Mangsa (15.3%). Among conventional farmers, it increases the probability of farmers to implement several adaptation strategies, including mixed cropping (15%), growing nonwater intensive crop (17.4%), crop rotation (21.8%), and changing irrigation techniques (15.2%).

#### 3.4.8. Farmer Group Membership

Farmers' membership in farmer groups has a positive and significant effect on several adaptation strategies selected by farmers. Organic farmers' access to farmer groups increases the probability of farmers to adopt particular adaptation strategies such as adjusting planting dates (38.7%) and using mulch (16.8%), while conventional farmers' access to farmer groups increases the probability of farmers to adopt some adaptation strategies including mixed cropping (16.2%), using superior varieties (18.9%), and crop rotation (11.8%).

#### 3.4.9. Access to Climate Training

Farmer's access to climate training does not significantly affect the adaptation strategies selected by organic and conventional vegetable farmers.

#### 3.4.10. Perceptions of Temperature Changes over the Last 30 years

Farmers who perceive that the air temperature increased over the last 30 years tend to have increased probability to implement several adaptation strategies such as growing nonwater intensive crops (16.9%), adjusting planting, and harvesting dates (15.9%) and increasing the uses of organic manure (17.7%). Among conventional farmers, it increases the probability to adopt several adaptation strategies such as mixed cropping (12.1%), adjusting planting and harvesting dates (20.7%), increasing the uses of organic manure (23.3%), and changing irrigation techniques (11%).

#### 3.4.11. Perceptions of Rainfall Changes over the Last 30 years

Farmers' perception that rainfall decreases has a positive effect on several adaptation strategies selected by farmers. Organic farmers who perceive that rainfall tends to decrease tend to have higher probability to implement particular adaptation strategies such as increasing the use of organic manure and using Pranata Mangsa by 15.2% and 17.2%, respectively. Among conventional farmers, it increases the probability to adopt several adaptation strategies such as using superior varieties (22%), increasing the uses of organic manure (25.2%), changing irrigation techniques (16.8%), and using Pranata Mangsa (31.6%).

## 4. Discussion

**Figure 3:**
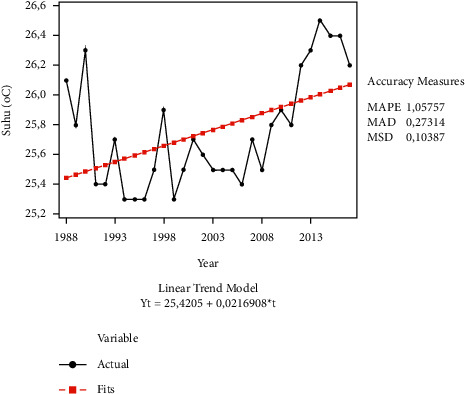
Temperature trend (°C) at research locations over the last 30 years (1988–2017) based on the linear trend model.

**Figure 4:**
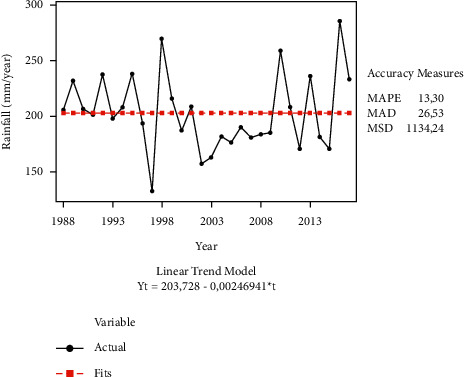
Rainfall trend (mm/year) at research locations over the last 30 years (1988–2017).

Our first analysis confirms that organic farmers have more accurate perceptions of climate change compared to conventional farmers. Organic vegetable farmers perceive that the temperature increased, and rainfall declined over the past 30 years. This perception is in line with the actual data from the Central Bureau of Statistics, where the average temperature over the past 30 years increased (Figure 3) and rainfall decreased over the past 30 years (Figure 4). This finding is in line with the findings of previous studies, stating that the majority of farmers perceive the occurrence of climate change, marked by an increasingly hot temperature and declined rainfall [16, 20, 45–49]. In addition, farmers who have experienced crop failure due to climate change such as drought or flooding will be more aware about climate change [50].

Organic and conventional vegetable farmers perceive that climate change has a negative impact on vegetable farming. Farmers' perceptions of the negative impacts of climate change on farming support the findings of previous studies [1, 7, 26, 51–53]. The three most significant impacts perceived by organic and conventional vegetable farmers are a decrease in the quality of yield, a decrease in production, and an increase in pests. The organic and conventional vegetable farmers interviewed mentioned that, in addition to rainfall and temperature changes, the occurrence of extreme weather in the study locations is more frequent. Heavy rain damages vegetables, thus lowering the quality of production. Prolonged drought in several areas has caused rainfed farming systems to experience crop failures.

Our second analysis confirms that the adaptation strategies implemented by organic vegetable farmers are more varied compared to those by conventional vegetable farmers. Organic vegetable farmers apply more adaptation strategies than conventional farmers [48]. Farmers more aware of climate change will implement more adaptation strategies to mitigate the effects of climate change [54–56]. The adaptation strategies most commonly implemented by organic farmers compared to conventional farmers are mixed cropping, crop rotation, shade, increasing the dose of organic manure, and changing irrigation techniques. Most organic farmers are bound by contracts in terms of sales with certain parties, so they make efforts by implementing various strategies to maintain the continuity of vegetable supply. Mixed cropping, crop rotation, shade, increasing the uses of organic manure, and changing irrigation techniques are the strategies that farmers believe to be able to support vegetable production for one year. During extreme rainfall or temperatures, shade will protect vegetables and reduce damage caused by extreme temperature and rainfall. Organic manure will support the soil by reducing the loss of water. A change is in irrigation techniques in the form of ponds as water storage for use during drought. Mixed cropping and crop rotation will maintain the availability of various types of vegetable and reduce the risks caused by climate change. Several previous studies show that some of the adaptation strategies implemented by farmers in dealing with climate change include mixed cropping, crop rotation [57], adjusting planting date [18, 29], increasing the use of organic manure [38], using shades [27], and changing irrigation techniques [57].

The adaptation strategies more widely adopted by conventional vegetable farmers are growing nonwater intensive crops, adjusting planting and harvesting dates, and using Pranata Mangsa. Pranata Mangsa is still widely used by conventional farmers. The conventional farmers interviewed stated that they grow vegetables by adjusting to the climate conditions, and they still use Pranata Mangsa to determine the planting dates and the most suitable commodities to be grown during that time. The results of this study support those of previous studies that farmers adapt by adjusting the growing seasons [29] and using Pranata Mangsa [57]. The adaptation strategies implemented by farmers aim to maintain agricultural production [58] and use it as a profitable opportunity [59].

Various types of adaptation strategies are implemented by farmers and influenced by climatic conditions, the types of farming, and other conditions such as political, economic, and institutional factors [29, 59]. Our third analysis confirms that age, education, experience, distance to extension services, access to credit, information about climate, and farmer groups as well as farmers' perceptions of temperature and rainfall changes over the past 30 years influence the climate change adaptation strategies selected by organic and conventional farmers.

Age, education, and experience will influence the selection of adaptation strategies. Age negatively affects the adaptation strategies selected by organic and conventional vegetable farmers. These results confirm the findings of a research conducted by [60] that young farmers more often adopt climate change adaptation strategies because they usually pay more attention to climate change. In fact, education may have either positive or negative impact on organic and conventional vegetable farmers' selection of climate change adaptation strategies. Some previous studies showed a significant positive relationship between farmers' education and climate change adaptation [61], but some also showed a negative relationship [62]. Organic farmers have higher education, so as to absorb technological innovations including innovations to adapt to climate change [48, 63]. Another factor influencing farmers' selection of adaptation strategies is experience. Organic and conventional vegetable farmers who have more farm experiences tend to be more aware of climate events in the past and better skill in assessing how to adjust their farming with extreme weather. A positive relationship between adaptation and experience is also shown by previous studies [21, 36, 64].

Farmers' access to extension services and input markets also influences farmers' adaptation strategies. Organic and conventional vegetable farmers' distance to extension services and input markets negatively affects farmers' selection of climate change adaptation strategies. The results of this study confirm those of previous studies, where distance to input market [28] and distance to extension services [31] negatively affect farmers' decision to adapt. The negative effect of distance to input markets on adaptation strategies is related to the limited access to input markets in terms of purchasing inputs on time [62]. Extension services and input markets should be easily accessed by farmers. Farmers who obtain information about climate conditions from extension services will have the knowledge of how to reduce climate change impacts as well as effective and efficient adaptation strategies [62], while input markets are far from their land, causing it difficult for them to access crop production inputs [31].

Farmer's access to credit, climate information, and farmer groups have a positive effect on the adaptation strategies selected by organic and conventional farmers. Farmers' access to credit will provide farmers with additional funding sources for the implementation of adaptation strategies. The finding of this study is in line with that of [22] that access to credit can increase farmers' opportunity to adapt. In addition, farmers who obtain climate information will have knowledge about climate, its impacts, and climate change adaptation in terms of vegetable farming. Farmers who often receive climate information will be more adaptable to climate change [15]. Farmer groups serve as a forum for farmers to search for information and technology [19]. Any information about mitigation and adaptation methods can be more effective if obtained from neighbours, peer groups, and other members of farmer groups [32]. Farmer group meetings at the research locations are held monthly, allowing farmers to obtain information regularly. In fact, farmers could obtain knowledge and information from farmer group meetings. Besides, farmers could also obtain climate change adaptation technologies from these meetings.

Farmers' perceptions of temperature and rainfall play a vital role in determining climate change adaptation strategies implemented by farmers. Perceptions will also determine the long-term measures that farmers will take in dealing with climate change. Organic farmers who perceive that the temperature is increasing will adapt by using organic manure. When organic farmers perceive that the rainfall is decreasing, they will increase the use of organic manure and use Pranata Mangsa.

On the other hand, conventional farmers who perceive that the temperature is increasing will make efforts to change their irrigation techniques, for example, by making reservoirs or drilled wells as the main source of water for farming. If the rainfall is decreasing, conventional farmers will use drought-and-flood-tolerant superior varieties, adjust planting and harvesting dates, increase the uses of organic manure to maintain the soil binding capacity, so the soil is not easily drying, change irrigation techniques by making reservoirs, and use Pranata Mangsa to determine both the suitable commodities and the planting times.

## 5. Conclusions and Recommendations

This research examined organic and conventional farmers' perception and adaptation to climate change and the factors that influence such decisions. Organic farmers' perception of temperature and rainfall over the past 30 years is in accordance with the climate data, indicating that organic farmers have more accurate perceptions of climate change compared to conventional farmers. Organic vegetable farmers perceive that climate change greatly affects vegetable farming. The three impacts experienced by most farmers are a reduction in the quality of crops, an increase in pests, and crop failure.

To reduce the impacts of climate change on vegetable farming, both organic and conventional farmers implement various adaptation strategies. The strategies include implementing mixed cropping, using superior varieties, growing nonwater intensive crops, implementing crop rotation, adjusting planting and harvest dates, increasing the use of organic manure, using shade, using mulch, changing irrigation techniques, and using Pranata Mangsa. In fact, the adaptation strategies implemented by organic farmers are more varied compared to those by conventional farmers. Organic farmers implement adaptation strategies to minimize the negative impacts of climate change on their farming, as a way for them to maintain the continuous supply of vegetables. In addition, farmers may select different strategies depending on the resources they have.

Policy makers and stakeholders shall contribute to increasing farmers' adaptive capacity in dealing with climate change by increasing farmers' access to climate information, input markets, credit, and farmer groups. In addition, policy makers and stakeholders shall provide more extension and information about climate and climate change adaptation strategies, particularly in relation to vegetable farming.

This study attempts to assess the perceptions and adaptations of organic and conventional farmers to climate change and analyse the factors that influence those decisions in Indonesia, using a logistic regression model. Data collected through self-administered questions, but some variables not included in this study, such as motivational factors, may have more influence towards the decision of farmers to further adapt. In addition, local wisdom regarding the planting season calendar in each research area should be considered in future research considering that these variables significantly affect the adaptation of farmers to climate change. The researcher also recommends that future work should consider other types of logistic regression.

## Figures and Tables

**Table 1:** Description of variables.

Variables	Description of variables
Age	Farmers' age (years)
Education	Farmers' education (years)
Farming experience	Farmers' experience in vegetable farming (years)
Distance to extension service	Distance to extension services (kilometres)
Distance to input markets	Distance to input markets (kilometres)
Access to credit	Dummy (1 = if farmers have access to credit institutions and 0 = otherwise)
Access to climate information	Dummy (1 = if farmers have access to climate information and 0 = otherwise)
Farmer group membership	Dummy (1 = if farmers join farmer group and 0 = otherwise)
Access to climate training	Dummy (1 = if farmers participate in climate trainings, and 0 = otherwise)
Perception of increased temperature	Dummy (1 = if farmers perceive that temperature increases over the last 30 years, and 0 = otherwise)
Perception of declined rainfall	Dummy (1 = if farmers perceive that rainfall decreases over the last 30 years and 0 = otherwise)

**Table 2:** Socioeconomic profile of respondent.

Characteristics	Organic		Conventional		Difference^a^
	Mean	Std. deviation	Mean	Std. deviation		
Age	45.214	12.836	45.455	11.527	−0.241	
Education	8.205	3.404	7.786	3.429	0.420	
Farming experience	19.902	13.283	22.259	12.876	−2.357	
Distance to extension service	3.632	3.171	3.057	1.400	0.575	^*∗*^
Distance to input markets	1.840	1.330	2.295	1.877	−0.456	^*∗∗*^
Access to credit	0.321	0.469	0.143	0.351	0.179	^*∗∗*^
Access to climate information	0.554	0.499	0.500	0.502	0.054	
Farmer group membership	0.929	0.259	0.625	0.486	0.304	^*∗∗∗*^
Access to climate training	0.107	0.311	0.080	0.304	0.027	
Perception of increased temperature	0.598	0.492	0.554	0.499	0.045	
Perception of declined rainfall	0.411	0.494	0.143	0.351	0.268	^*∗∗∗*^

^a^Significance based on Pearson chi-square for differences in proportions between the two groups or *t*-test for the average difference between the two groups. ^*∗∗∗*^Significant at 1% level. ^*∗∗*^ Significant at 5% level. ^*∗*^Significant at 10% level.

**Table 3:** Organic and conventional farmers' perceptions of climate change impacts on vegetable farming.

Impact	Organic (%)	Conventional (%)	Difference^a^	
It is difficult to predict growing season	59.821	52.679	7.143	
Irregular planting pattern	50.000	50.893	−0.893	
Increase in pests	66.964	78.571	−11.607	^*∗∗*^
Decrease in production	75.000	74.107	0.893	
Production failure	53.571	55.357	−1.786	
Reduction in the quality of crops	75.893	71.429	4.464	
Increase in crop failure risks	65.179	70.536	−5.357	
Increase in loss risks	66.071	70.536	−4.464	
Decrease in water supply	23.214	30.357	−7.143	

^a^Significance based on Pearson chi-square for differences in proportions between the two groups. ^*∗∗*^Significant at 5% level.

**Table 4:** Climate change adaptation strategies of organic and conventional vegetable farmers in Java.

Adaptation strategies	Organic (%)	Conventional (%)	Difference^a^	
Implementing mixed cropping	95.536	85.714	9.821	^*∗∗*^
Using superior varieties	91.071	87.500	3.571	
Growing nonwater intensive crops	48.214	50.893	−2.679	
Implementing crop rotation	95.536	88.393	7.143	^*∗∗*^
Adjusting planting and harvesting dates	53.571	62.500	−8.929	^*∗∗*^
Increasing the use of organic manure	44.643	22.321	22.321	^*∗∗∗*^
Using mulch	92.857	91.071	1.786	
Using shade	34.821	8.929	25.893	^*∗∗∗*^
Changing irrigation techniques	37.500	11.607	25.893	^*∗∗∗*^
Using Pranata Mangsa	35.714	48.214	−12.500	^*∗*^

^a^Significance based on Pearson chi-square for the differences in proportions between the two groups. ^*∗∗∗*^Significant at 1% level. ^*∗∗*^Significant at 5% level. ^*∗*^Significant at 10%.

**Table 5:** Estimation coefficients, marginal effects, and partial elasticity in logistic analysis of factors influencing organic vegetable farmers' adaptation strategies.

Variable	Expected sign	Adaptation strategies																			
			y1		y2		y3		y4		y5		y6		y7		y8		y9		y10	
Age	+/−	a	0.018		0.080		−0.004		0.018		−0.035		0.002		−0.029		0.033		−0.017		−0.087	^*∗∗*^
		b	0.000		0.006		−0.001		0.000		−0.006		0.000		−0.002		0.006		−0.004		−0.015	
		c	0.035		0.290		−0.091		0.035		−0.737		0.058		−0.096		0.966		−0.491		−0.653	
Education	+/−	a	−0.019		0.239		−0.218	^*∗∗*^	−0.019		−0.165	^*∗∗*^	0.132	^*∗*^	−0.012		0.133	^*∗∗*^	0.066		−0.157	^*∗∗*^
		b	−0.000		0.017		−0.042		−0.000		−0.030		0.025		−0.001		0.025		0.014		−0.026	
		c	−0.009		0.147		−0.028		−0.009		−0.694		0.554		−0.007		0.679		0.322		−0.869	
Farming experience	+/−	a	0.225		−0.075		−0.014		0.225		0.005		−0.011		0.071		−0.024		0.004		0.083	^*∗∗*^
		b	0.006		−0.005		−0.003		0.006		0.001		−0.002		0.004		−0.005		0.001		0.014	
		c	0.104		−0.141		−0.152		0.104		0.042		−0.126		0.071		−0.314		0.046		0.031	
Distance to extension service	−	a	0.816		−0.170	^*∗*^	−0.103		0.816		−0.125	^*∗*^	0.032		0.600		−0.031		−0.105		0.057	
		b	0.021		−0.012		−0.020		0.021		−0.023		0.006		0.032		−0.006		−0.023		0.009	
		c	0.106		−0.076		−0.198		0.106		−0.224		0.064		0.094		−0.078		−0.256		0.118	
Distance to input markets	−	a	−1.777	^*∗*^	−0.005		−0.620	^*∗∗*^	−1.777	^*∗*^	−0.414	^*∗∗*^	−0.075		0.354		−0.413	^*∗∗*^	−0.042		−0.380	^*∗*^
		b	−0.046		−0.000		−0.120		−0.046		−0.075		−0.014		0.019		−0.078		−0.009		−0.063	
		c	−0.222		−0.001		−0.480		−0.222		−0.312		−0.078		0.037		−0.559		−0.051		−0.386	
Access to credit	+	a	−0.952		−0.853		−0.225		−0.952		−0.277		1.328	^*∗∗*^	−1.114		−0.506		0.720		0.238	
		b	−0.025		−0.061		−0.043		−0.025		−0.050		0.253		−0.059		−0.096		0.154		0.039	
		c	−0.017		−0.030		−0.040		−0.017		−0.544		0.130		−0.040		−0.104		0.122		0.051	
Access to climate information	+	a	4.052	^*∗*^	−0.107		0.091		4.052	^*∗*^	−1.476	^*∗∗*^	0.722		0.017		0.627		−0.598		0.919	^*∗*^
		b	0.106		−0.008		0.018		0.106		0.266		0.138		0.001		0.119		−0.128		0.153	
		c	0.036		−0.007		0.024		0.036		0.488		0.181		0.001		0.213		−0.219		0.287	
Farmer group membership	+	a	0.375		1.225		0.140		0.375		2.144	^*∗*^	0.002		3.173	^*∗∗*^	−0.053		0.020		0.634	
		b	0.010		0.088		0.027		0.010		0.387		0.000		0.168		−0.010		0.004		0.105	
		c	0.013		0.098		0.067		0.013		0.001		0.001		0.142		−0.032		0.012		0.373	
Access to climate training	+	a	−3.259		0.838		1.215		−3.259		0.130		−0.304		0.992		0.502		−0.092		−1.328	
		b	−0.085		0.060		0.235		−0.085		0.023		−0.058		0.052		0.095		−0.020		−0.221	
		c	−0.029		0.007		0.054		−0.029		0.861		−0.014		0.089		0.022		−0.006		−0.107	
Perception of increased temperature	+	a	2.402		−1.501		0.873	^*∗*^	2.402		0.879	^*∗*^	0.928	^*∗∗*^	0.222		−0.418		0.583		0.648	
		b	0.063		−0.108		0.169		0.063		0.159		0.177		0.012		−0.079		0.125		0.108	
		c	0.043		−0.107		0.234		0.043		0.282		0.257		0.099		−0.172		0.198		0.231	
Perception of declined rainfall	+	a	0.223		0.377		0.475		0.223		0.103		−0.796	^*∗*^	−0.141		−0.766		−0.344		1.037	^*∗∗*^
		b	0.006		0.027		0.092		0.006		0.019		−0.152		−0.007		−0.146		−0.074		0.172	
		c	0.004		0.010		0.089		0.004		0.019		−0.206		−0.050		−0.233		−0.092		0.241	

y1, implementing mixed cropping; y2, using superior varieties; y3, growing nonwater intensive crops; y4, implementing crop rotation; y5, adjusting planting and harvesting dates; y6, increasing the uses of organic manure; y7, using mulch; y8, using shade; y9, changing irrigation techniques; y10, using Pranata Mangsa. ^*∗∗*^, ^*∗*^Significant at 5% level and 10% level. ^a^Estimation coefficients, ^b^marginal effects, and ^c^partial elasticity.

**Table 6:** Estimation coefficients, marginal effects, and partial elasticity in logistic analysis of factors influencing conventional farmers' adaptation strategies.

Variable	Expected sign	Adaptation strategies																			
			y1		y2		y3		y4		y5		y6		y7		y8		y9		y10	
Age	+/−	a	−0.101	^*∗∗*^	0.077		−0.052	^*∗∗*^	−0.074	^*∗*^	−0.080	^*∗∗*^	−0.014		0.034		−0.059		−0.025		−0.043	
		b	−0.008		0.006		−0.011		−0.005		−0.013		−0.002		0.002		−0.004		−0.002		−0.008	
		c	−0.680		0.446		−0.210		−0.394		−0.487		−0.491		0.123		−0.424		−0.993		−0.013	
Education	+/−	a	0.025		0.209	^*∗*^	0.017		0.074		−0.140	^*∗*^	−0.213	^*∗∗*^	−0.119		0.012		0.200	^*∗*^	−0.194	^*∗*^
		b	0.002		0.015		0.004		0.005		−0.022		−0.026		−0.008		0.001		0.015		−0.035	
		c	0.029		0.184		0.065		0.078		−0.461		−0.312		−0.083		0.081		0.272		−0.868	
Farming experience	+/−	a	0.106	^*∗∗*^	0.094	^*∗∗*^	0.019		0.126	^*∗∗*^	0.041		0.005		0.013		0.010		−0.013		0.049	^*∗*^
		b	0.009		0.007		0.004		0.009		0.007		0.001		0.001		0.001		−0.001		0.009	
		c	0.278		0.318		0.208		0.226		0.356		0.086		0.021		0.196		−0.253		0.515	
Distance to extension service	−	a	−0.875	^*∗∗*^	−0.197		0.074		−0.759	^*∗∗*^	−0.298		0.318		0.225		−1.003	^*∗∗*^	0.421		0.051	
		b	−0.073		−0.014		0.016		−0.055		−0.048		0.039		0.016		−0.075		0.032		0.009	
		c	−0.285		−0.097		0.106		−0.186		−0.334		0.725		0.060		−0.621		0.075		0.071	
Distance to input markets	−	a	−0.598	^*∗∗*^	−0.291		0.212		−0.517	^*∗∗*^	−0.657	^*∗∗*^	−0.164		−0.327	^*∗*^	−0.337		0.004		0.499	
		b	−0.050		−0.021		0.045		−0.038		−0.105		−0.020		−0.023		−0.025		0.000		0.090	
		c	−0.233		−0.112		0.198		−0.135		−0.421		−0.297		−0.098		−0.711		0.008		0.440	
Access to credit	+	a	1.612		0.104		0.721		0.130		−0.271		−0.750		1.590	^*∗*^	1.306		0.992		0.394	
		b	0.134		0.008		0.154		0.009		−0.043		−0.092		0.110		0.098		0.075		0.071	
		c	0.014		0.002		0.045		0.002		−0.022		−0.080		0.043		0.095		0.097		0.028	
Access to climate information	+	a	1.801	^*∗∗*^	−0.332		0.818	^*∗*^	2.997	^*∗∗*^	−0.818		1.036		1.522		−1.134		2.016	^*∗∗*^	−0.399	
		b	0.150		−0.024		0.174		0.218		−0.130		0.127		0.105		−0.085		0.152		−0.072	
		c	0.177		−0.027		0.256		0.294		−0.205		0.361		0.027		−0.532		0.900		−0.125	
Farmer group membership	+	a	1.949	^*∗∗*^	2.579	^*∗*^	−0.240		1.626	^*∗*^	0.870		0.443		0.095		−0.094		1.481		0.636	
		b	0.162		0.189		−0.051		0.118		0.139		0.054		0.007		−0.007		0.112		0.115	
		c	0.122		0.299		−0.079		0.102		0.186		0.202		0.004		−0.051		0.754		0.199	
Access to climate training	+	a	−0.276		0.063		−0.103		−0.115		1.389		0.521		−0.872		0.984		0.460		0.218	
		b	−0.023		0.005		−0.022		−0.008		0.221		0.064		−0.060		0.074		0.035		0.039	
		c	−0.005		0.001		−0.006		−0.002		0.037		0.028		−0.008		0.072		0.033		0.012	
Perception of increased temperature	+	a	1.455	^*∗*^	−1.254		−0.172		0.495		1.302	^*∗∗*^	1.901	^*∗∗*^	0.259		0.478		1.451	^*∗*^	−0.309	
		b	0.121		−0.092		−0.037		0.036		0.207		0.233		0.018		0.036		0.110		−0.056	
		c	0.091		−0.123		−0.049		0.031		0.349		0.696		0.014		0.214		0.635		−0.091	
Perception of declined rainfall	+	a	−1.546		−3.005	^*∗∗*^	−0.311		−1.266		0.981		2.052	^*∗∗*^	0.690		0.000		2.228	^*∗∗*^	1.742	^*∗∗*^
		b	−0.128		−0.220		−0.066		−0.092		0.156		0.252		0.048		0.000		0.168		0.316	
		c	−0.028		−0.134		−0.024		−0.023		0.061		0.147		0.006		0.002		0.139		0.093	

y1, implementing mixed cropping; y2, using superior variety; y3, growing nonwater intensive crops; y4, implementing crop rotation; y5, adjusting planting and harvesting dates; y6, increasing the uses of organic manure; y7, using mulch; y8, using shade; y9, changing irrigation techniques; y10, using Pranata Mangsa. ^a^Estimation coefficients, ^b^marginal effects, and ^c^partial elasticity.

## Data Availability

The data used to support the findings of this study are available from the corresponding author upon request.
